# A Design Method of Traveling Wave Rotary Ultrasonic Motors Driving Circuit under High Voltage Using Single-Sided Hertzian Contact Forced Oscillator Model

**DOI:** 10.3390/mi14010064

**Published:** 2022-12-26

**Authors:** Xiaoniu Li, Tianlu Huang, Ning Zhao, Youtao Shen, Jiada Huang, Xuan Li, Jiayi Li, Lin Yang

**Affiliations:** 1 State Key Laboratory of Mechanics and Control of Mechanical Structures, Nanjing University of Aeronautics and Astronautics, Nanjing 210016, China; 2 Shanghai Aerospace Control Technology Institute, Shanghai 201108, China; 3 Centre for Medical and Industrial Ultrasonics, James Watt School of Engineering, University of Glasgow, Glasgow G12 8QQ, UK

**Keywords:** ultrasonic motor, piezoelectric material, nonlinear vibration

## Abstract

Traveling wave rotary ultrasonic motors (TRUMs) are widely used in various industrial processes due to their attractive features, such as compact structure, high accuracy, and fast response. However, the major limiting factors of the operational performance of TRUMs under high-voltage excitation are the nonlinear behavior caused by the nonlinearities of the piezoelectric materials and the friction between the stator and rotor of the motor. In this study, a nonlinear dynamics model and an identification method are presented to directly design the driver circuit for suppressing the nonlinear behavior under high voltage excitation. Firstly, by studying the time–frequency characteristics of the isolated electrode voltage, a single-sided Hertzian contact forced oscillator model of TRUMs is established, involving the nonlinearities of the piezoelectric material and friction. Secondly, a harmonic balance nonlinear identification is proposed in the time domain for TRUMs. The influence of the voltage and preload on the nonlinear phenomena is discussed. Lastly, a novel driver circuit is proposed to suppress the nonlinearities using feedback from the isolated electrode. Experiments showed that the total harmonic distortion decreased by 89.4% under 500 Vpp. The proposed drive circuit design method is used to find a high excitation voltage and preload to achieve greater motor output power.

## 1. Introduction

Traveling wave rotary ultrasonic motor is one of the most commonly used ultrasonic motors, presenting advantages of compact structure, high accuracy, fast response, no electromagnetic interference, and self-locking [1,2]. This technology is now being more widely used in precision engineering [3], biomedical engineering [4], and robotics [5]. One of the common problems of an ultrasonic motor is its nonlinear behaviors, which presents as a result of the intrinsic nonlinearity of the piezoelectric material [6] as well as the contact between the stator and rotor of the motor, especially at high voltage excitation [7]. For example, the jump phenomenon [8] of the motor will introduce difficulties in the precision adjustments of the resonant frequency and motor speed. Further, if the motor is operated at a frequency in the unstable region of the amplitude-frequency characteristic, systematic divergences will occur. The nonlinear behavior of the motor is challenging the efficiency of the system [9], the design of the driving circuit [10,11], and the controller [12]. Therefore, it is crucial to study the nonlinear dynamic response of ultrasonic motors to develop an effective method to design the drive circuit.

It has been reported in numerous literature that the nonlinear behavior of the piezoelectric material is the major contributing factor to the nonlinearity of ultrasonic motors. At high excitation levels, the hysteresis of the piezoelectric material-induced nonlinearity has resulted in a jump phenomenon in the amplitude–frequency characteristic of the vibration of the ultrasonic motor, as thoroughly studied by Petit et.al [13]. To model a longitudinal torsional hybrid type of ultrasonic motor, the cubic term of the torsional stiffness has been considered by Nakagawa et.al [14]. Cao et.al [15] considered the nonlinearities of the piezoceramic material and established a nonlinear dynamics model of the TRUM, whose transient characteristics are strongly affected by the creep effects at the friction interfaces. The above models all add a cubic non-linear term to the linear model and can reflect the non-linear vibration of the structure under high-voltage excitation. However, it is difficult to reflect the non-linearity problem caused by contact in this way.

Viscous and Coulombic frictions are believed to exist in the dynamic contact between the stator and rotor, as suggested by Wallaschek et.al [16]. The motor velocity essentially depends on the friction coefficient, which has resulted in a stick-slip effect at the contact zone, based on the study of the surface roughness, pre-pressure, sliding speed, plastic deformation, and contact zone wear [17]. Li et.al [18] discovered that a pure stick motion could cause a nonlinear dead-zone behavior in motors, strictly hindering the design of motor controls. Zhu et.al [19] investigated the dead zone of the vibration amplitude by simplifying the contact model to static friction. Zhang et.al [20] developed the Dahl model to avoid discontinuities of contact forces in both left and right directions of motion for V-shape linear ultrasonic motors. In addition, the dead zone [21], wear of the friction interface [22], and change of pre-pressure [23] are also frequently related to contact friction [24]. The friction exacerbates the nonlinearity of the vibration of the stator in an ultrasonic motor, which has led to more complex nonlinear dynamics and has further complicated the nonlinear problems of TRUM as a whole. Currently, nonlinear studies of ultrasonic motors are mainly focused on a single cause, and dynamic modeling and analyses of multiple nonlinear causes have not been comprehensively investigated. The above models are all numerical models, so it is difficult to use them to drive and control motors directly.

Due to piezoelectric material nonlinearities and contact problems, a drive and control system design is challenging. To ensure a high-performance operation of the ultrasonic motors, a drive, and control system is the key, which in most cases, is implemented by the vibration control strategy [25]. To investigate the comprehensive performance of an ultrasonic motor, the driving frequency should be selected at an anti-resonance point of the nano-conductor, as suggested by Zhao et.al [1]. Devos et.al [26] analyzed the thermal–electromechanical coupling model of a motor whose driving frequency was slightly higher than the resonant frequency to dissipate the heat generated in the motor. Using the mechanical Q factor analysis model and the admittance phase experimental model of the ultrasonic motor, Shi et.al [27] further determined that an optimal driving frequency should be the average of the frequency with the maximum conductance and the frequency with the maximum resistance. Currently, the mainstream frequency-tracking method is based on the solute voltage of the isolated electrode. Rather than tracking the resonant frequency directly, Shi et.al [28] developed an optimal frequency-tracking scheme that considers the input voltage’s phase angle as the control variable. However, the above ultrasonic motor drive control method faces severe challenges for application in ultrasonic motors [29]. When the phase differences between the current and voltage are used to achieve frequency tracking, it is difficult to ensure that the phase differences remain constant, which could cause serious incidents, such as reversals of motors, because of the multiple values of the nonlinear vibration phase.

This paper is dedicated to a further improvement of the operational performance of TRUM by developing a nonlinear dynamic numerical model to predict the nonlinearity in experiments and an identification method to analyze the dynamic characteristics of the system. Upon this, a novel driving strategy implemented in hardware configurations is proposed to restrain the nonlinearity. Experimental results have validated the effectiveness of the proposed scheme.

## 2. Analysis of Nonlinear Dynamics

### 2.1. Mechanism of TRUM

**Figure 1:**
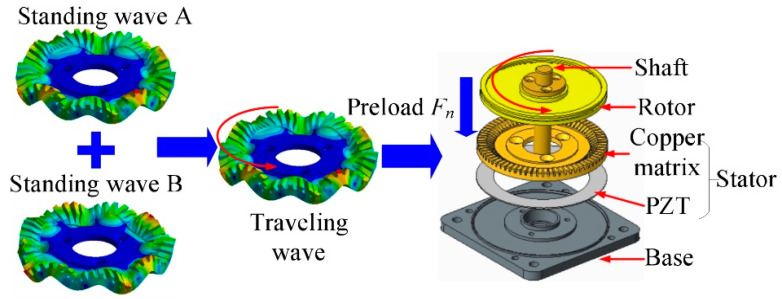
Schematic of the working principle [30].

The design concept of TRUM was first proposed by Sashida et.al [30] and the working principle of TRUM is shown in Figure 1. To generate a rotating traveling wave, two-phase orthogonal standing waves have been excited. The piezoceramic ring was divided into several sectors and was polarized individually. The positive (+) and negative (−) signs illustrate the polarization directions of the sectors, which are divided into two groups, A and B. A one-quarter wavelength λ/4 sector and three-quarters of wavelength 3λ/4 sector were created to separate these two groups, forming an isolated electrode to provide an auto-frequency tracking signal [31].

Due to the traveling wave in the stator, the ac voltage generated by the isolated electrode because of the positive piezoelectric effect can be calculated from [1]:

(1) $$v(t)=\frac{{\kappa W}_{t}}{C_{0}}\cos\omega \;t$$

where κ is the force coefficient of the piezoceramic material, *W_t_* is the amplitude of the vibration displacement, $C_{0}$ is the clamping capacitance of the isolated electrode, and *ω* is the angular excitation frequency. The generated ac voltage signal of the isolated electrode has the same frequency as the excitation signal, and the amplitude of the generated voltage is proportional to that of the traveling wave of the stator, making it convenient to obtain the characteristics of the stator vibration by analyzing the generated voltage from the isolated electrode.

### 2.2. Frequency Response Analysis of TRUM

**Figure 2:**
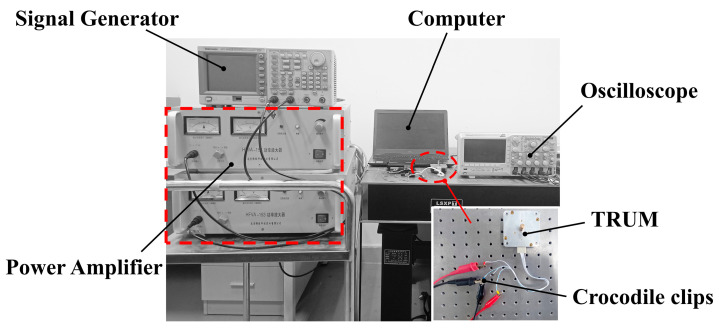
Schematic diagram of the experimental setup.

Experiments have been carried out to study the nonlinear vibration characteristics of TRUM. The experimental setup is presented in Figure 2, consisting of a signal generator (AFG3022, Tectronix Inc., Beaverton, OR, USA), a power amplifier (HFVP-153, Foneng Inc., Nanjing, China), an oscilloscope (DPO2014, Tectronix Inc., Beaverton, OR, USA), a TRUM system (NUAA Super Control Technologies Co., Ltd., Nanjing, China), and a computer. The preload of the motor was set to 400 N. A sine wave signal was generated by the signal generator and then amplified and matched by the power amplifier and matching circuit before being applied to the ultrasonic motor. The generated voltage from the isolated electrode was monitored and recorded using the oscilloscope, and the fast Fourier transform (FFT) of the signal was calculated.

**Figure 3:**
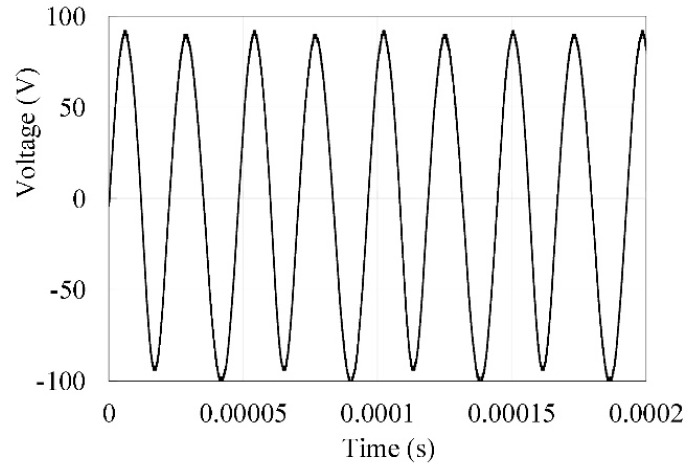
Generated voltage from the isolated electrode.

Figure 3 presents the time domain signal of the voltage generated from the isolated electrode when the stator’s bending (B-09) mode was excited at a 41.5 kHz frequency and a 500 Vpp voltage input to the piezoceramic ring.

**Figure 4:**
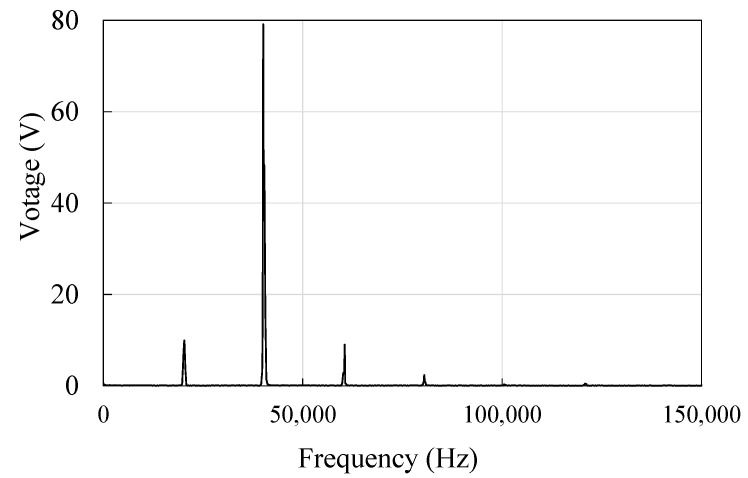
Voltage spectrum of the isolated electrode.

Applying FFT to the time domain signal produces the frequency response, shown in Figure 4. The curve suggests that the frequency components at 1/2, 3/2, and 2nd to the fundamental frequency of the generated voltage from the isolated electrode are pronounced, with the fundamental frequency component having the highest power, followed by the 1/2 and 3/2 components, which demonstrate a similar level, and the 2nd harmonic frequency component whose power is the lowest, in that order.

**Figure 5:**
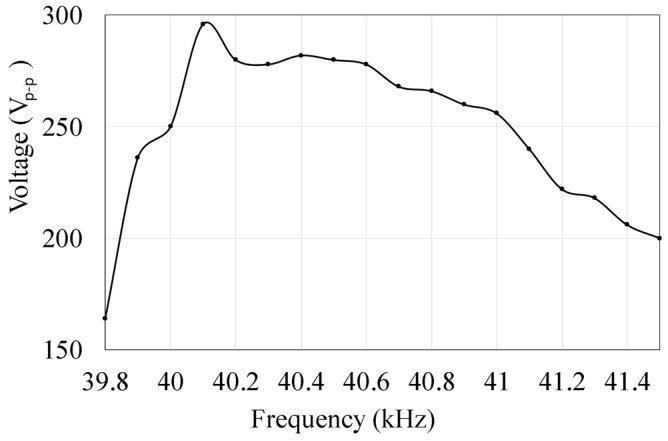
Frequency response of the generated voltage from the isolated electrode.

Next, we investigate the frequency response of the generated voltage from the isolated electrode under a constant voltage excitation, sweeping from below to above the resonant frequency, as shown in Figure 5. The generated voltage from the isolated electrode reached the peak when the motor’s excitation signal approached the resonant frequency of the system at around 40.1 kHz.

**Figure 6:**
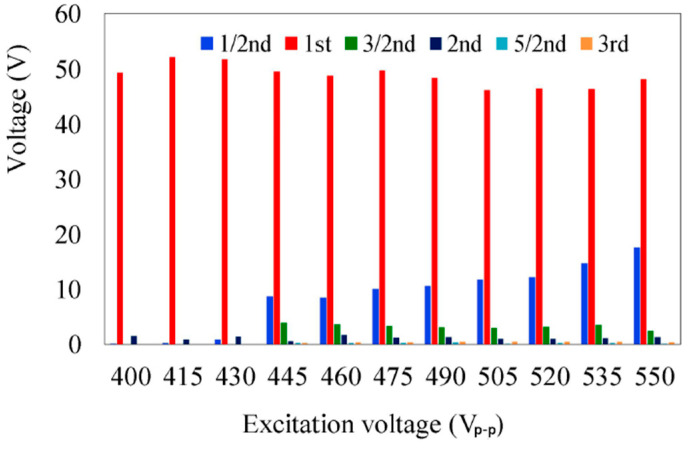
Relationship between the harmonic components of the isolated electrode and excitation voltage.

The harmonic components of the soliton voltage under different excitation voltages of the motor at a 39.5 kHz excitation frequency are shown in Figure 6. The generated voltage of the non-primary resonance harmonic components has increased with the increase in the applied voltage. However, the primary resonance voltage presents a fluctuating trend, which indicated that the energy had been transferred to the harmonic components.

**Figure 7:**
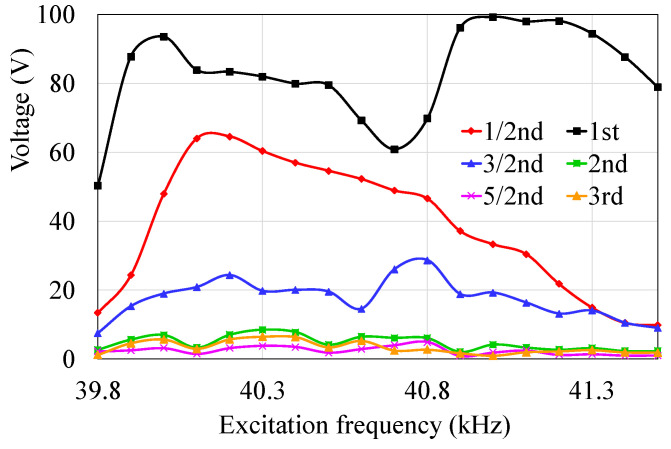
Relationship between the harmonic components of the generated voltage from the isolated electrode and excitation frequency.

The generated voltage of the harmonic components at different excitation frequencies is shown in Figure 7. There is a decrease in the non-primary resonance harmonic components.

## 3. Modeling and Identification

**Figure 8:**
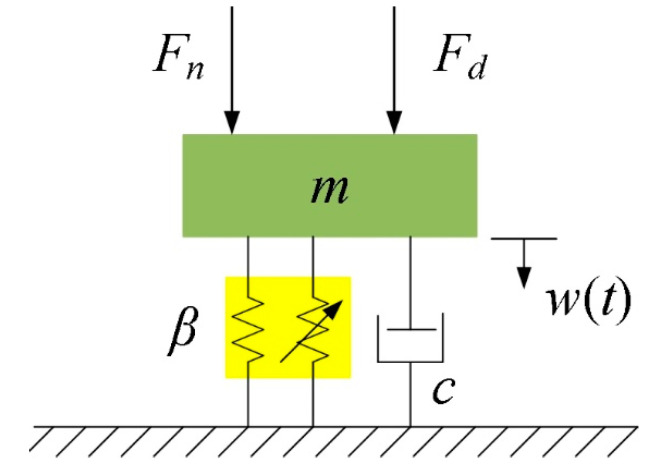
Simplified model of TRUM.

Considering the contact between the stator and the rotor, and the nonlinearity of the piezoceramic material, a single-sided Hertzian-contact forced oscillator model can be employed to represent this system [32]. The TRUM could be modeled as a spring-mass-damper-lumped system of a single degree of freedom with a nonlinear spring arranged in parallel, as shown in Figure 8.

Assuming Hertz’s law of contact, the nonlinear restoring force can be developed from the material properties and the contact geometry, and its equation of motion can be written as:

(2) $$m\ddot{w}(t)+c\dot{w}(t)+\beta w^{\frac{3}{2}}{(t)=F}_{n}{+F}_{d}$$

where

(3) $$\begin{array}{c} F_{n}=N\left({1+k}_{c}\cos\omega \;t\right)\\ F_{d}{=\mathrm{AV}}_{\mathrm{pp}}\cos\Omega \;t \end{array}$$

Here, *N* is the preload of the TRUM; *k_c_* is the dynamic contact force coefficient of the contact layer between the stator and rotor; *A* is the force coefficient of the piezoelectric element; *V_pp_* is the amplitude of the excitation voltage; *w* is the vibration displacement of the stator; *Ω* is the excitation voltage frequency; and *β* is the nonlinear coefficient to be identified.

The assembled motor is not convenient for testing the stator vibration and is also not convenient for real-time control of the driving circuit. Since the voltage of the isolated electrode of the ultrasonic motor reflects the vibration of the stator [1], we found a corresponding nonlinear phenomenon in the voltage of the isolated electrode in Section 2. Therefore, the voltage of the isolated electrode can be used to identify model parameters.

Using the relationship between the voltage *v(t)* of the isolated electrode and the stator displacement *w*(*t*), Equation (2) can be written as follow:

(4) $$m\ddot{v}{(t)+c\dot{v}(t)+\beta v}^{\frac{3}{2}}{(t)=F}_{n}{+F}_{d}$$

Next, we identified the model parameters. Firstly, the linear equivalent parameters *m* and *c* was measured by an impedance analyzer (Agilent 4294A, Agilent Technologies Japan, Ltd., Hyogo, Japan) using a 500 mV excitation voltage. The values of *m* and *c* were 0.0577809 kg and 1 N s/m, respectively. Then, the harmonic balance nonlinear identification method [33,34] was used to identify the parameters through the generated voltage signal of the isolated electrode in the time domain.

The Fourier series expansion of the generated voltage of the isolated electrode is as follows:

(5) $$\begin{array}{l} v\left(t\right)=\sum_{n=-\infty }^{+\infty }a_{n}\cdot e^{\mathrm{in}\omega t}\\ \dot{v}\left(t\right)=\sum_{n=-\infty }^{+\infty }{\mathrm{ik}\omega a}_{n}\cdot e^{\mathrm{in}\omega t}\\ \ddot{v}\left(t\right)=\sum_{n=-\infty }^{+\infty }{-k}^{2}\omega^{2}a_{n}\cdot e^{\mathrm{in}\omega t} \end{array}$$

The Fourier expansion of the nonlinear part is given by:

(6) $$v^{\frac{3}{2}}\left(t\right)=\sum_{n=-\infty }^{+\infty }b_{n}e^{\mathrm{in}\omega t}$$

The Fourier expansion of the excitation function is:

(7) $$q\left(t\right){=F}_{n}{+F}_{d}=\sum_{n=-\infty }^{+\infty }c_{n}\cdot e^{\mathrm{in}\omega t}$$

where *a_n_*, *b_n_*, and *c_n_* are constants.

Substituting Equations (5)–(7) to Equation (2) produces:

(8) $${-n}^{2}\omega^{2}a_{n}{+2\mu \mathrm{in}\omega a}_{n}+(\frac{\beta }{m}{)b}_{n}{=c}_{n}$$

where *µ* = *c*/2*m*.

Taking the real part, the expression that identifies the nonlinear parameter can be calculated as:

(9) β=∑−NNRe(an)n2ω2+2μnωIm(an)+Re(cn)Re(L1)∑−NNRe(L1)

**Figure 9:**
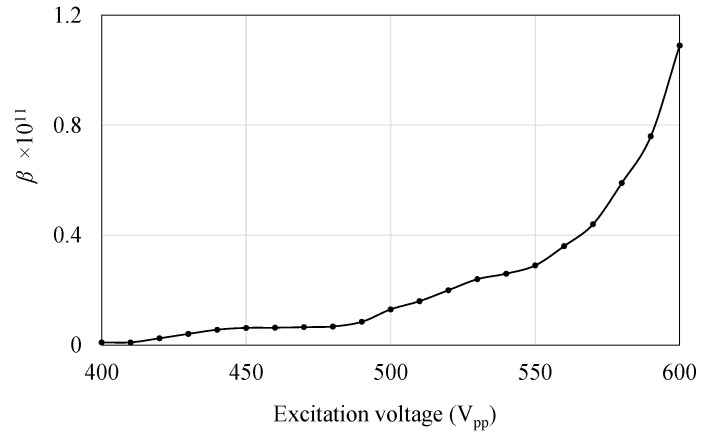
Value of nonlinear parameters with the excitation voltage.

The value of the nonlinear parameter *β* as the change in the excitation voltage is shown in Figure 9. The nonlinear parameter *β* has increased steadily until 500 Vpp and then soared to around 1.1 at around 600 Vpp.

**Figure 10:**
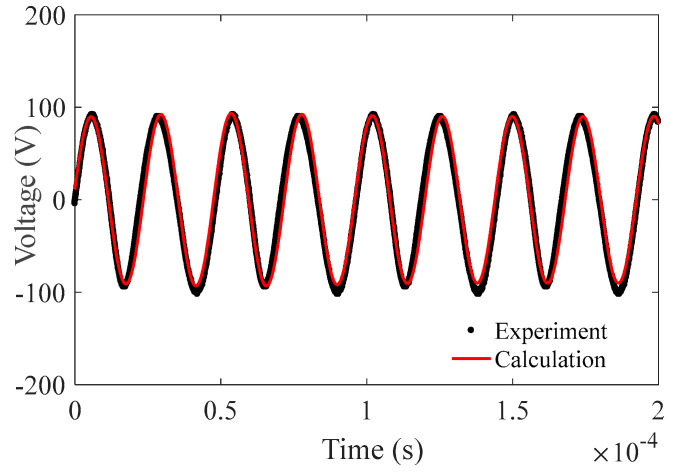
Comparison between the modeled results and experimental measurements.

Figure 10 shows the comparison of the voltage components of the simulated results and the experimental measurements. The prediction and experiments share a good agreement concerning the waveform, amplitude, and phase.

To further analyze the nonlinear behavior of the TRUM, we used a multi-scale method, which allows us to solve the amplitude-frequency response curve of this nonlinear system. A study on impacting the Hertzian contact [35] concludes that the solution of amplitude *r* and phase *θ* at the steady-state motion of this nonlinear system must satisfy the following conditions:

(10) $$\left\{\begin{array}{c} \frac{\mathrm{dr}}{\mathrm{dt}}{=\mathrm{Ar}+H}_{1}{\sin\theta +H}_{2}\cos\theta \\ r\frac{d\theta }{\mathrm{dt}}{=\mathrm{Br}+\mathrm{Cr}}^{3}{+H}_{1}{\cos\theta -H}_{2}\sin\theta \end{array}\right.$$

where

(11) $$\begin{array}{l} A=-\frac{\alpha }{2},B=-\frac{\lambda }{2\bar{\omega }}-\frac{\alpha^{2}}{8\;\bar{\omega }}-\frac{\beta G}{\;\bar{\omega }}-\frac{\lambda^{2}}{{8\;\bar{\omega }}^{3}}\\ C=-\left(\frac{{5\beta }^{3}}{{12\;\bar{\omega }}^{3}}+\frac{3\gamma }{8\;\bar{\omega }}\right){,H}_{1}=\frac{\lambda \sigma }{{8\;\bar{\omega }}^{3}}-\frac{\sigma }{2\;\bar{\omega }}\\ H_{2}=\frac{\alpha \sigma }{{8\;\bar{\omega }}^{2}},\alpha =2\xi ,\xi =\frac{c}{2\mathrm{mv}}\\ G=\frac{\beta }{2}{\left(\frac{\;\bar{a}}{{\;\bar{\Omega }}^{2}}\right)}^{2},\;\bar{\omega }=\frac{\omega }{v},\;\bar{\Omega }=\frac{\Omega }{v}\\ v^{2}=(\frac{3k_{0}}{2m})Z_{s}^{\frac{1}{2}},Z_{s}={\left(\frac{F_{n}}{\beta }\right)}^{\frac{2}{3}} \end{array}$$

By eliminating *θ*, the system amplitude–frequency response equation can be derived as:

(12) $$C^{2}r^{6}{+2\mathrm{BCr}}^{4}+\left(A^{2}{+B}^{2}\right)r^{2}-\left(H_{1}^{2}{+H}_{2}^{2}\right)=0$$

**Figure 11:**
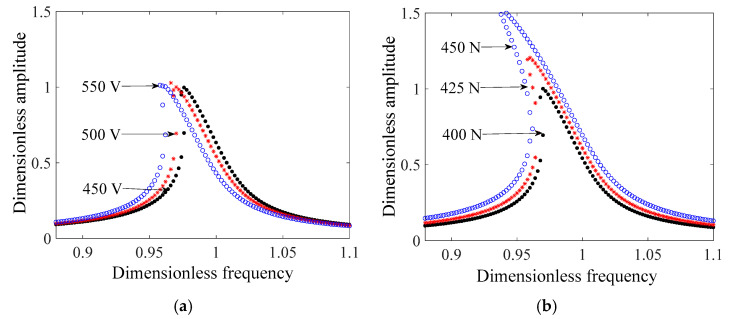
Frequency response characteristics of systems. (**a**) Frequency response characteristics under different voltage; (**b**) Frequency response characteristics under different preloads.

The system amplitude and frequency characteristics under different excitation and different preload can be obtained using Equation (12), and the results are shown in Figure 11.

**Figure 12:**
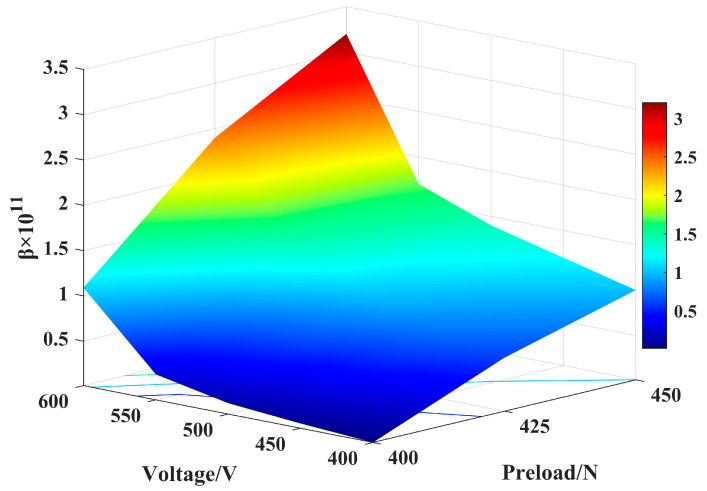
Value of nonlinear parameters with the excitation voltages and preloads.

In general, the amplitude–frequency curves present a jump phenomenon near the resonant frequency, and the backbone curves exhibit a soft spring characteristic. In Figure 11a, as the excitation voltage increases, the shift of the resonant frequency of the system increases. From Figure 11b, the nonlinearity has presented an increasing trend with the increase in the preload, and the shift in the resonant frequency is the greatest. compared to the excitation voltage effect on the mechanical output of the system, a higher preload has led to a significant increase in the vibration amplitude, as shown in Figure 12. This means that it is crucial to properly adjust the value of the preload in the motor assembly due to this high sensitivity.

## 4. Drive Circuit Design Method and Experiment Validation

**Figure 13:**
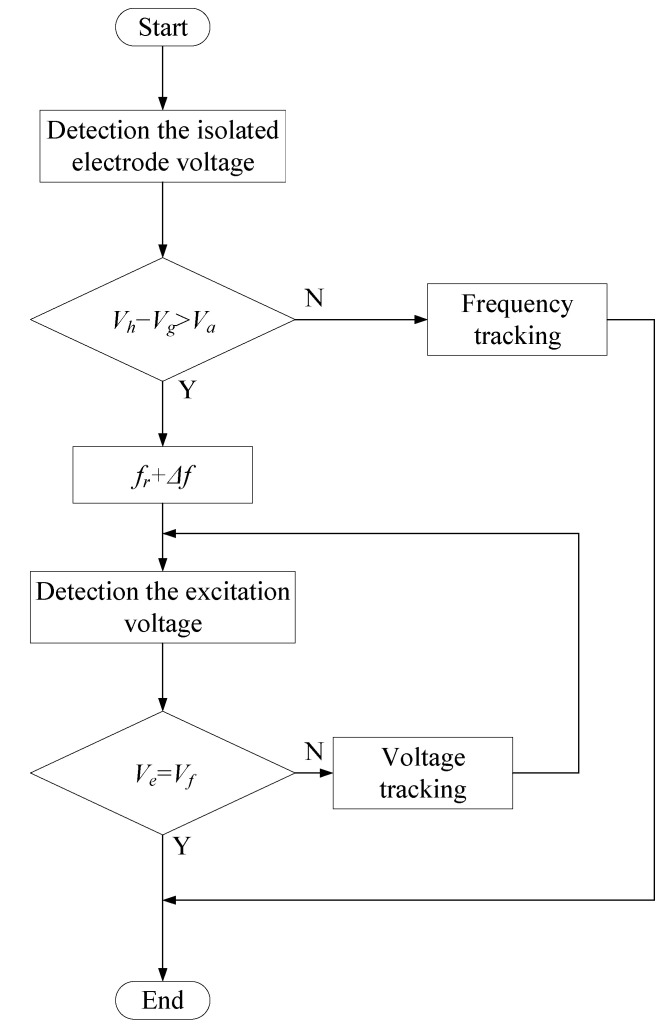
Flow diagram of the proposed method.

From the above analysis, it is understood that the isolated electrode of the ultrasonic motor is a piezoelectric sensor placed on the stator, and the isolated electrode voltage can fully reflect the material’s nonlinearity and friction. To suppress the nonlinearity of the motor, we propose a method to automatically adjust the excitation frequency and the applied voltage based on the isolated electrode voltage, as shown in Figure 13. This method uses the difference between the generated voltage of the harmonic components *V_h_* and the fundamental component *V_g_* of the isolated electrode to control the excitation frequency. When the difference between *V_h_* and *V_g_* is greater than a prescribed value *V_a_*, the excitation frequency increases and the system escapes the nonlinear region. When the voltage *V_e_* applied to the ultrasonic motor is equal to the set voltage *V_f_*, the bus voltage of the power amplifier circuit is adjusted to achieve a constant voltage driving.

**Figure 14:**
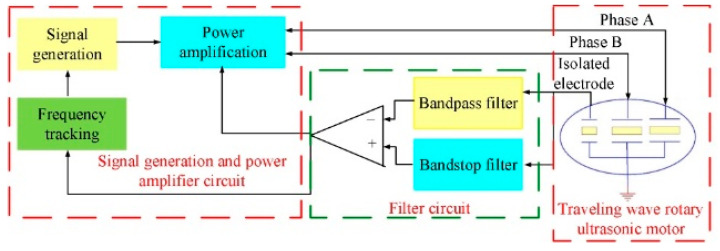

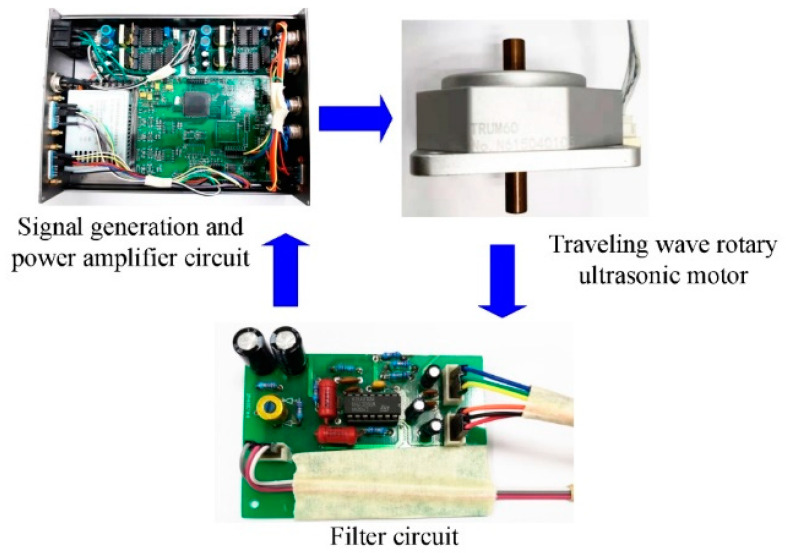
Block diagram and fabricated circuits using the proposed method.

The experimental diagram to validate the effectiveness of the proposed strategy is shown in Figure 14. The driving circuit for suppressing the system’s nonlinearity mainly consists of a signal generator, a filter circuit, a power amplifier, and a frequency tracking circuit. The generated voltage from the isolated electrode of the motor passes through a band-pass filter and a band-stop filter is used to provide the effective values of the fundamental component and the harmonic components of the voltage, respectively. Thereafter, the effective values are compared to calculate the difference, which is used to control the amplitude of the excitation voltage of the motor.

**Figure 15:**
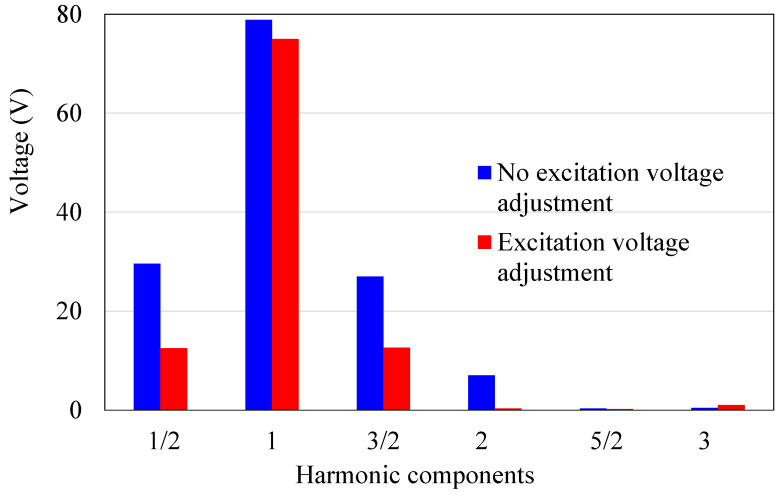
Harmonic components of the isolated electrode voltage with and without the proposed method.

Experimental results are presented in Figure 15 using a 500 Vpp input voltage. The harmonic components at 1/2, 3/2, and 2nd to the fundamental frequency of the generated voltage are greatly reduced after the implementation of the novel driving circuit under 500 Vpp. The experiments showed that the total harmonic distortion decreased by 89.4%. The proposed circuit has caused a considerable reduction in the generated voltage of the isolated electrode for the harmonic components, although the fundamental frequency component also demonstrates a slight drop, as shown in Figure 15. As a result, the motor is ensured to operate outside of the nonlinear region, so that the jump phenomenon can disappear, and the nonlinearity can be effectively suppressed.

**Figure 16:**
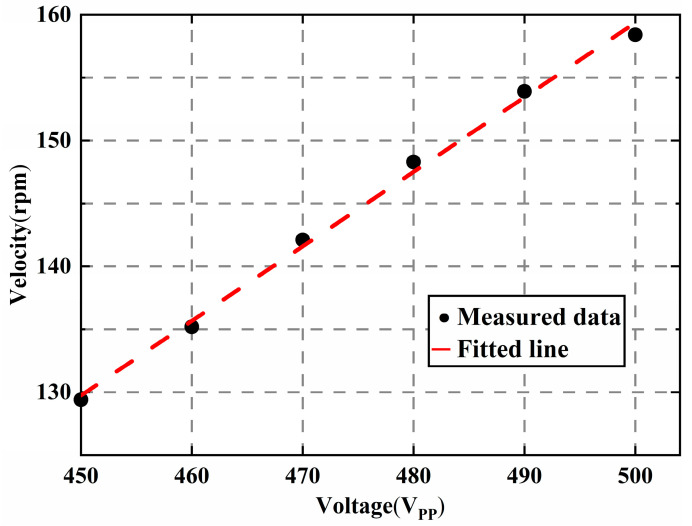
Velocity at different excitation voltage with the proposed method.

**Figure 17:**
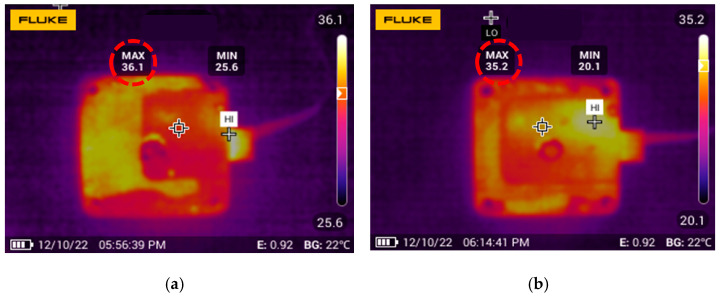
Comparison of generated heat of the TRUM. (**a**) Generated heat without the proposed method; (**b**) Generated heat with the proposed method.

The experimental results about the speed and the generated heat are also presented in Figure 16 and Figure 17. The rotation speed of the motor is measured at high voltages with the suppression system. The measured range of the excitation voltage is between 450 Vpp and 500 Vpp, and the interval is 10 V. The output speed of the motor is almost linear about the voltage even at high voltages. A pocket thermal camera (PTi120, FLUKE, Everett, WA, USA) is used to measure the generated heat. The motor worked continuously for ten minutes under 500 Vpp, and the images of the temperature distribution were taken using the thermal camera. Experiments showed that the max temperature was reduced to 0.9 °C.

## 5. Conclusions

To design traveling wave rotary ultrasonic motor drive circuits under high-voltage excitation, a complete scheme including a dynamic model, a time-domain identification method, and a novel driving strategy has been proposed. When the fundamental generated voltage harmonic component of the isolated electrode was excited under high-voltage excitation, the unwanted sub-harmonic and super-harmonic components were also generated, contributing significantly to the nonlinearity of TRUM. The single-sided Hertzian-contact forced oscillator model was established, consisting of a nonlinear spring and a preload. The generated voltage of the isolated electrode was used to directly identify the values of the nonlinear parameters in the time domain. The simulated values predicted by the numerical model show a good agreement with experimental measurements. The first-order primary resonance of the system was analyzed using a multi-scale method, and the amplitude–frequency response equation of the system was also obtained. Results showed that the nonlinear phenomenon of the system appears to be more outstanding when increasing the preload and the amplitude of the excitation voltage. The output speed is almost linear about high excitation voltage, and the generated heat of the motor can be reduced. The method for adjusting the excitation frequency and voltage of the motor by using the difference between the harmonic components and the fundamental component of the isolated electrode was successfully implemented to restrain the nonlinearities under high-voltage excitation. The proposed drive circuit design method is used to find a high excitation voltage and preload to achieve greater motor output power.

## Data Availability

Not applicable.
